# Isolation and identification of *Desmodium* root exudates from drought tolerant species used as intercrops against *Striga hermonthica*

**DOI:** 10.1016/j.phytochem.2015.06.026

**Published:** 2015-09

**Authors:** A.M. Hooper, J.C. Caulfield, B. Hao, J.A. Pickett, C.A.O. Midega, Z.R. Khan

**Affiliations:** a Department of Biological Chemistry and Crop Protection, Rothamsted Research, Harpenden, Hertfordshire AL5 2JQ, UK; b International Centre of Insect Physiology and Ecology, P.O. Box 30772, Nairobi, Kenya

**Keywords:** *Desmodium uncinatum*, *Desmodium intortum*, *Desmodium incanum*, *Desmodium ramosissimum*, *Striga hermonthica*, Parasitic weeds, Root exudate, Di-*C*-glycosylflavone, *Striga* inhibition

## Abstract

Plants from the genus *Desmodium*, in particular *D. uncinatum*, are used on sub-Saharan small-holder farms as intercrops to inhibit parasitism of cereal crops by *Striga hermonthica* and *Striga asiatica via* an allelopathic mechanism. The search for *Desmodium* species which are adapted to more arid conditions, and which show resilience to increased drought stress, previously identified *D. intortum*, *D. incanum* and *D. ramosissimum* as potential drought tolerant intercrops. Their potential as intercrops was assessed for resource poor areas of rain-fed cereal production where drought conditions can persist through normal meteorological activity, or where drought may have increasing impact through climate change. The chemical composition of the root exudates were characterised and the whole exudate biological activity was shown to be active in pot experiments for inhibition of *Striga* parasitism on maize. Furthermore, rain fed plot experiments showed the drought tolerant *Desmodium* intercrops to be effective for *Striga* inhibition. This work demonstrates the allelopathic nature of the new drought tolerant intercrops through activity of root exudates and the major compounds seen in the exudates are characterised as being *C*-glycosylflavonoid. In young plants, the exudates show large qualitative differences but as the plants mature, there is a high degree of convergence of the *C*-glycosylflavonoid exudate chemical profile amongst active *Desmodium* intercrops that confers biological activity. This defines the material for examining the mechanism for *Striga* inhibition.

## Highlights

• Root exudates from drought tolerant *Desmodium* spp. were analysed by HPLC and LCMS.
• Compounds were identified as di-*C*-glycosylflavones using standards.
• After 4 months, exudates from drought tolerant species were similar to each other.
• Exudates possessed *Striga* inhibitory properties in pot experiments in all cases.
• *Desmodium* spp. identified are active against *Striga* in semi-arid field conditions.

## Graphical abstract

Drought tolerant *Desmodium* species, when mature, produce root exudates that inhibit *Striga* parasitism and which comprise a range of di-*C-*glycosylflavones.

**Figure:**
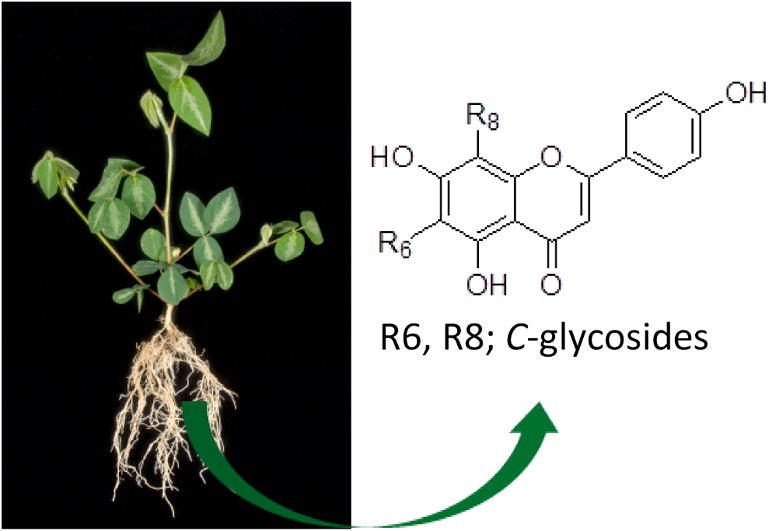

## 1 Introduction

In subsistence farming regions of sub-Saharan Africa (SSA), where parasitic weeds of the *Striga* genus devastate subsistence cereal crops, a successful intervention called push–pull technology effectively inhibits *Striga* damage and has been adopted by more than 96,000 farmers (http://www.push-pull.net/, Fischler, 2010). The technology utilises forage legumes in the *Desmodium* genus (Fabaceae) as an intercrop which provides the key chemical components for inhibiting development of *Striga* in the field. The mechanism by which this occurs has been shown to be caused by allelopathic root exudates from *Desmodium* that both stimulate the germination of *Striga*, (Khan et al., 2002) but more importantly inhibit the subsequent development of the germinated seed, (Tsanuo et al., 2003; Hooper et al., 2010) so that in the field, almost no parasitism is observed (Khan et al., 2010; Pickett et al., 2010). The technology blends in with the cultural practise of mixed cropping and has been taken up by farmers introduced to it through their peers in farmer-led groups (Khan et al., 2008, 2014; Murage et al., 2011). It provides additional benefits to insect pest (cereal stemborer) and weed control by providing fodder for zero-grazed livestock while improving soil quality through organic carbon and fixed N (Khan et al., 2008a; Midega et al., 2013). In order to expand the technology into areas that are either more arid or under threat of increased drought through climate change, new species of *Desmodium* must be identified that are resilient to drought and which can also provide the chemistry that confers inhibition of *Striga*. Once identified, the intercrop should protect drought tolerant cereal crops such as sorghum or new drought tolerant maize varieties against *Striga*, thereby increasing food security for small-holder farmers. In addition, the further characterisation of active root exudate chemistry provides the basis for identifying the enzymic and genetic basis for allelochemical production *in planta* (Hooper et al., 2009; Khan et al., 2008b).

In our screening for drought tolerance, three *Desmodium* species were originally identified as showing potential as they survived extended periods of drought when grown in African screen-house conditions. They are *Desmodium intortum* (Mill.) Urb., *Desmodium incanum* (G. Mey.) DC. and *Desmodium ramosissimum* G. Don. We hypothesise that taxonomically related *Desmodium* species may produce similar root exudate chemistry to the *Desmodium* species known to be effective in the field (*Desmodium uncinatum* (Jacq.) DC.), and so be potential intercrops for inhibition of *Striga* parasitism of subsistence cereals in more arid agronomic environments,. In order to investigate their potential for *Striga* inhibition, the root exudates of these plants were collected and analysed by HPLC and liquid chromatography-mass spectrometry (LCMS) to determine whether the same *Striga* inhibiting chemistry previously identified from *D.* uncinatum (Hooper et al., 2010) was present and which could be attributed to inhibition of *Striga*. In addition, by performing screen-house pot experiments, the drought tolerant *Desmodium* plant root exudates were tested against *Striga hermonthica* on maize, to demonstrate *Striga* inhibiting biological activity. The drought tolerant *Desmodium* species were then tested as *Striga* inhibiting intercrops for sorghum in rain fed plot experiments under field conditions in Western Kenya.

## 2 Results and discussion

### 2.1 *Striga* inhibition by drought tolerant *Desmodium* species root exudates

**Fig. 1:**
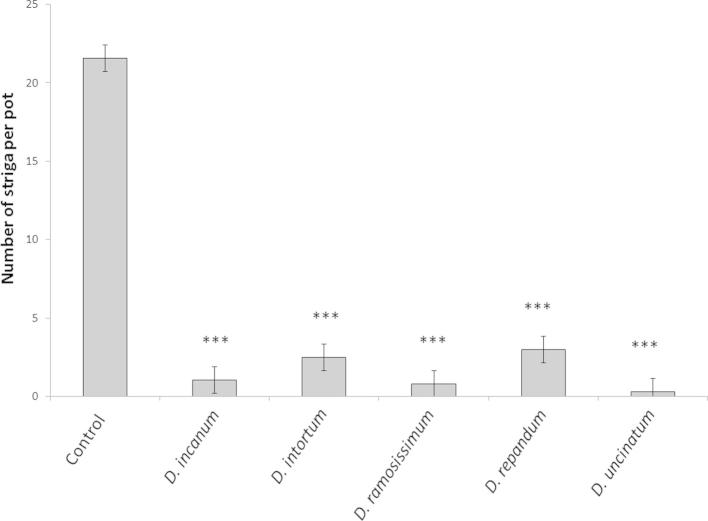
Mean (±SE) number of *S. hermonthica* per pot treated with *Desmodium* root exudates (*n* = 18). Asterisks indicate significant difference (*P* < 0.001) from the control treatment.

In screen house experiments, the parasitism of maize by *S. hermonthica* was inhibited strongly by irrigation with the root exudates of all the tested *Desmodium* species compared with irrigation through soil alone (Fig. 1). The mean number of *Striga* plants emerged on root exudate treated maize differed among treatments with distilled water control (Fig. 1; *F*_5,107_ = 98.23, *P* < 0.001) showing the root exudates for all tested *Desmodium* species were effective in inhibiting *Striga* parasitism.

### 2.2 *Striga* inhibition by drought tolerant *Desmodium* species in demonstration plots

**Fig. 2:**
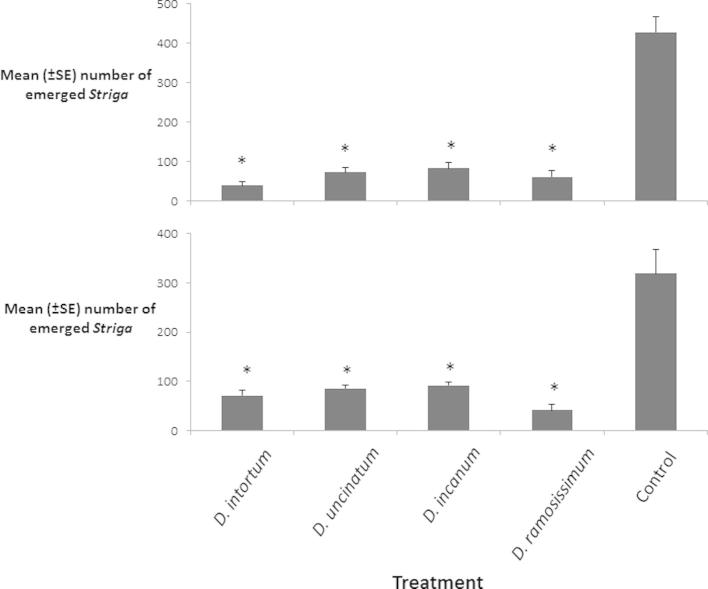
Mean (±SE) number of emerged *S. hermonthica* plants per plot during the short rain (above) and long rain (below) seasons of 2014 in western Kenya. Within a graph, asterisk indicates a significant difference to control (*P* < 0.05).

The four species of Desmodium for which exudates were studied, were grown as intercrops with drought tolerant sorghum (the early maturing commercial hybrid Gadam Hamam) in rain fed plots on the icipe Thomas Odhiambo Campus on the shores of Lake Victoria. The performance of the plots after the short and rainy seasons of 2014 were assessed (Fig. 2). All the drought tolerant *Desmodium* species tested showed significant *Striga* inhibiting properties in the field, as they did in the pot experiments, with no significant differences to the performance of *D. uncinatum* the intercrop now used widely in farmer fields.

### 2.3 Exudate analysis

**Fig. 3:**
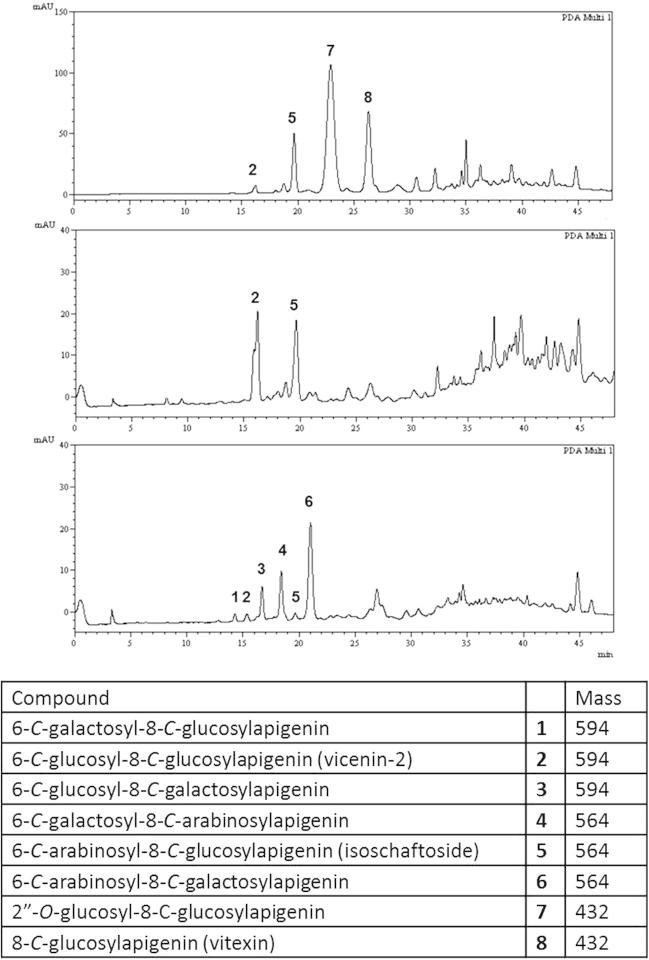
HPLC analysis of exudates from *D. uncinatum* (top), *D. intortum* (middle) and *D. incanum* (bottom) collected for 1 week, 4–5 weeks after germination with LCMS data elucidating the structure of *C*-glycosylflavonoid components. UV detection was monitored at 350 nm.

The root exudates of *D. uncinatum*, *D. intortum* and *D. incanum* were collected one month after the transfer of plants into hydroponic solution and analysed by HPLC (Fig. 3). The seedlings were transferred to the hydroponic solution at two weeks after germination from seeds. The structure of the major flavonoid peaks in these initial exudates were identified by HPLC co-elution with known standards, isolated from extracting leaf or root tissue of conspecific plants which were fully characterised by nuclear magnetic resonance (NMR) spectroscopic and electrospray ionisation mass spectrometry (ESIMS) analysis (Fig. 3). The major components seen in the *C*-glycosylflavonoid (CGF) region of *D. uncinatum* are isoschaftoside (**5**), vitexin (**8**) and a broad signal for 2″-*O*-glucosylvitexin (**7**). In the case of *D. intortum*, the major components are vicenin-2 (**2**) and isoschaftoside (**5**). The component isoschaftoside (**5**) has been reported previously as being able to interfere with *Striga* development post-germination (Hooper et al., 2010). However, analysis of the exudate from *D. incanum* revealed that it comprised a number of CGFs structures, of which isoschaftoside was only a minor component. All three species were found to produce isoschaftoside (**5**) in their exudate in varying quantity but there are very clear differences in the compounds that comprise the exudate at this stage in plant development.

**Fig. 4:**
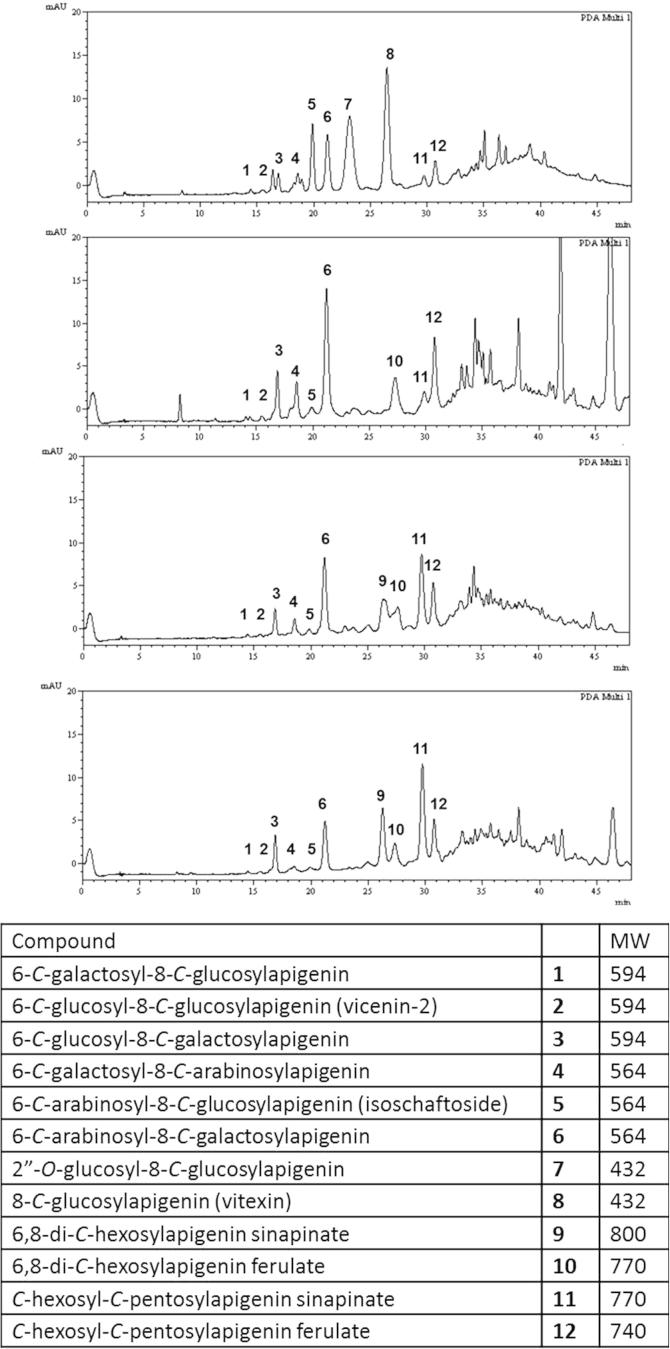
HPLC analysis of exudates (top to bottom) from *D. uncinatum*, *D. intortum, D. incanum* and *D. ramosissimum* collected for 1 week after 4 months in hydroponics with LCMS data elucidating the structure of *C*-glycosylflavonoid components. UV detection was monitored at 350 nm.

The same three species *D. uncinatum*, *D. intortum*, *D. incanum* along with another species, *D. ramosissimum*, which was added to the experiment subsequently because it was found to regenerate after an extended period of drought (>2 months), were grown in hydroponics for four months and the root exudates collected again for HPLC and LCMS analysis. At this time of plant growth, although some differences still existed in the chemical make-up of the exudates, there was now a high degree of similarity as demonstrated in Fig. 4. The many differences observed in young plant root exudates were now reduced and the root exudates matched closely. After HPLC analysis and tentative identification of the flavonoid components through retention time, LCMS analysis generated their molecular weight and their fragmentation was used to verify the nature of attachment of hexose or pentose moieties to the flavonoid aglycone. LCMS experiments using higher cone voltages showed fragmentation of the metabolites which generated losses of 120 and 90 Da, typical of *C*-linked hexoses, and losses of 90 and 60 Da typical for *C*-linked pentoses. This identified the hexose or pentose moieties seen in the molecules to be both *C*-linked. The structures were then identified by co-elution with standards obtained and fully characterised from conspecific plant tissues. Specific chemicals identified and present in each of the four *Desmodium* species plant root exudates are labelled and shown in Fig. 4. The presence of di-*C*-glycosylflavones coupled to phenolic acids was not detectable in the early stages of exudate production and were also not detected in the tissues of the plants extracted for natural product standards. They are presumably exuded shortly after they are biosynthesised, implying an important role in rhizosphere chemical ecology yet to be elucidated. The absence in *Desmodium* plant tissue means no chemical standard could be fully structurally elucidated and the position of the ester and the position and type of hexose and pentose remain targets for characterisation.

### 2.4 Chemical characterisation

Compounds that were isolated from tissues are shown in Table 1. These were subsequently used to verify the structures of the chemicals in the plant exudates by HPLC co-elution and LCMS analysis. Compounds **1**, **3**, **4** and **6** were isolated from *D. incanum* root tissue, compound **2** from *D. incanum* leaf tissue and compounds **5**, **7** and **8** from *D. uncinatum* root tissue. The *C*-linked sugar moieties show restricted rotation in NMR experiments and without recording NMR data at very high temperature, rotamers or broadened peaks were observed. In the case of **3**, two major rotamers could not be coalesced at 350 K in methanol, but interconverted at 290 K and all rotamers were shown to be stable on the NMR timescale at 243 K. Both major rotamers were therefore characterised at 290 K.

## 3 Conclusions

Allelochemicals generated from the root exudates of plants in cropping systems possess the potential for delivering desirable biological activities in both low input and high-input agriculture as they may be delivered in the desired site for activity (Hooper et al., 2010; McNally et al., 2003; Macias et al., 2008, 2009). Our initial screens of desmodium species performed to test for tolerance of extended periods of drought resulted in three species being taken forward to examine whether they can act as potential intercrops for cereal production The root exudates of the drought tolerant intercrop species *D. intortum*, *D. incanum* and *D. ramosissimum* are similar to each other and to *D. uncinatum* after these plants have developed in hydroponic solution for four months. While all plants produce isoschaftoside, an allelochemical previously identified as inhibitory to post-germination *Striga* development, over time, a blend of di-*C*-glycosylflavones are exuded by all the species examined when maturing. This class of compounds has been reported to possess various ecological and biological activities such as anti-microbial activity (McNally et al., 2003), mycorrhizal colonisation stimulants of melon root (Akiyama et al., 2002), antifeedant activity (Chul-sa et al., 2009; Byrne et al., 1996), and activity antagonistic to *Striga* (Hooper et al., 2010) as well as many other medicinal activities (Talhi and Silva, 2012). The root exudate blends characterised here demonstrated *Striga* inhibition in pot experiments and so provide the chemical basis for intervention against *Striga* inhibition under rain fed field conditions. Demonstrating *Striga* inhibition in research field plots by drought tolerant *Desmodium* in semi-arid rain fed agriculture shows that the field potency predicted by screen house pot experiments was achieved. The application of these plants as intercrops with drought tolerant cereals, such as sorghum, in participating farmer fields is now to be tested. *Desmodium* root exudate activity in arid conditions rather than hydroponics, where moisture in the rhizosphere may effect allelochemical production or movement may not be important as there must be sufficient moisture in the ground to grow cereal crops. In the research plots here under semi-arid conditions, the data show the exudates are effective. In addition, the *Desmodium* intercrop is perennial and produces exudates year round, so while leaching due to an instance of heavy rainfall may reduce soil concentrations, they can be replenished over time. The identification of root exudate metabolites also allows the creation of synthetic root exudates or blends containing one or more components, to probe further the chemical basis of *Striga* inhibition by *Desmodium*. The drought tolerant species have demonstrated resilience to the arid conditions where push–pull technology can make an impact in the yield of staple cereals for small-holder farmers. Climate change is also expected to increase drought stress in existing areas where the technology has been successful to date, making the identification and characterisation of new plants possessing the required ecological chemistry vital to protect crops against the biotic stress of parasitic weed damage.

## 4 Experimental

### 4.1 Plant material

Seeds of *D. uncinatum*, *D. intortum*, *D. incanum* and *D. ramosissimum* were obtained from the seed bank at the International Livestock Research Institute (Addis Ababa, Ethiopia) and grown in seed multiplication plots at the field station of the International Centre for Insect Physiology and Ecology, Thomas Odhiambo Campus (icipe-TOC) on the shores of Lake Victoria in western Kenya.

### 4.2 Pot experiments

Screen house trials were conducted at icipe-TOC using methodologies adapted from Khan et al., 2002. The allelochemical activity of *Desmodium* intercrops against *S. hermonthica* parasitism on maize was tested by irrigating maize grown in *Striga* infested soil with an aqueous solution of chemical components, eluting from established *Desmodium* plants. Pots (20 cm) containing the drought tolerant desmodium species, *D. intortum*, *D. repandum*, *D. incanum* and *D. ramosissimum*, along with *D. uncinatum* as a positive control and soil alone as a negative control, were placed on shelves and received distilled water at a rate of 1.25 ml/min, thus allowing the flow of water by gravity through the *Desmodium* root mass and into pots containing maize situated below. These pots contained a *Striga*-susceptible maize variety (WH505) planted in soil which had been inoculated with approximately 3000 *S. hermonthica* seeds/pot. Autoclaved soil was used in all experiments, and no additional nitrogen was applied. Emergence of *S. hermonthica* parasites on maize were monitored in all treatments.

### 4.3 Statistical analysis

The number of emerged *Striga* plants was analysed using one-way analysis of variance with blocking (batch) in GenStat 16. Fischer’s least significant tests were used to compare means. There were six pots in three batches to give 18 total replicates.

### 4.4 Striga inhibition by drought tolerant Desmodium species in demonstration plots

Studies were conducted at Thomas Odhiambo Campus of the International Centre of Insect Physiology and Ecology (icipe), Mbita, located along the eastern shores of Lake Victoria, western Kenya (0°25′S, 34°12′E). Field trials were conducted during the long and short rainy season of 2014 to investigate effectiveness of the various *Desmodium* species in suppression of *S. hermonthica*. During this calendar year, rainfall was approximately 900 mm. Field plots measured 6 m by 6 m, and were arranged in a complete randomized design in four replications. The treatments comprise sorghum intercropped with each of the four *Desmodium* species and a monocrop. Sorghum was planted at a row-to-row distance of 60 cm and a plant-to-plant distance of 30 cm within a row. The sorghum variety used was the *S. hermonthica* susceptible, early maturing commercial hybrid Gadam Hamam, recommended for mid-altitude regions. Sorghum was planted at an inter-row spacing of 75 cm and an intra-row spacing of 30 cm, while *Desmodium* was planted through a drilling system in furrows between the rows of sorghum. The number of emerged *Striga* plants was counted from 45 plants per plot from within a radius of 15 cm around the base of each sorghum plant, and data expressed as the number of emerged *Striga* per plot. The observation was made at 10 weeks following sorghum emergence since *Striga* emergence often peaks at about 10 weeks of crop emergence in the region (Midega et al., 2013).

### 4.5 HPLC analysis of exudates

*Desmodium* seeds were germinated in a sandy soil and grown until approximately 4 cm high so that a significant root system had established. Subsequent removal of the plants was facilitated by the high sand content. The plants were washed and placed in a hydroponic solution (40% Long Ashton) in plastic tubs containing about 2 litres for approximately 50 plants. During 1 week the hydroponic solution was circulated at 5 mL/min using a peristalsis pump through a Pasteur pipette containing XAD-4 Amberlite polymer to trap out organic material. The trap was washed with MeOH (25 mL) and the solvent removed. The residue was dissolved in 1 mL MeOH for HPLC analysis on a Shimadzu VP series HPLC system using an ACE AQ C-18 column (250 mm x 4.6 mm, 5 μm). The mobile phase A (5% HCO_2_H in H_2_O) and B (MeOH) used a gradient program at 1 mL/min, initially 95:5 (A:B), to 85:15 at 3 min, 75:25 at 13 min, 70:30 at 25 min, 45:55 at 35 min, 45:55 at 45 min, 5:95 at 46 min, 5:95 at 58 min, 95:5 at 60 min (Ferreres et al., 2003).

### 4.6 LCMS analysis of exudates

The exudates isolated as described in Section 4.5, evaporated to dryness and re-dissolved in 1 mL methanol, were subject to LCMS analysis using the Micromass Quattro Ultima bench top triple quadrupole mass spectrometer attached to Waters Acquity UPLC system (Ultra Performance Liquid Chromatography). The mass spectrometer was operated in negative ion mode, with a capillary voltage of 2.7KV, cone voltage 50–180 eV, mass range 50–1000 *m*/*z*. Source temperature 130 °C, desolvation temperature 350 °C, desolvation gas flow 1000 L/h (nitrogen) and cone gas flow 60 L/h (nitrogen). Where required, selected ions were admitted to the collision cell for MSMS analysis with argon admitted at a pressure of 2.1 e^−3^ mbar, causing CID. Samples were injected *via* the Acquity sample manager, injecting 1 μl onto an Acquity UPLC BEC HSS C18 1.8 μm 2.1 × 150 mm column. Run time was 63 min at a flow rate of 0.4 mL/min. Solvents used are defined A (water, 0.05% formic acid) and B (methanol). The mobile phase used a gradient program, initially 95:5 (A:B), to 85:15 at 3 min, 75:25 at 13 min, 70:30 at 25 min, 45:55 at 35 min, 45:55 at 40 min, 5:95 at 46 min, 5:95 at 58 min, 95:5 at 60 min, 95:5 at 63 min.

### 4.7 Isolation of compounds **1**–**8** from Desmodium roots and leaf tissue

Tissue from the desmodium species (5–20 g) of interest was dropped into liquid nitrogen. The frozen tissue was then ground in a mortar and pestle until powdered and then blended in a solution of 75% MeOH in H_2_O (100 mL). The extract was passed through a glass sinter and evaporated to dryness by rotary evaporation. The residue was dissolve in a 5–10 mL of 25% MeOH in H_2_O and centrifuged at 10,000 rpm to remove insoluble material. The supernatant was decanted and subjected to purification on a Shimadzu VP series HPLC system using an ACE AQ C-18 column (250 mm × 10 mm, 5 μm). The mobile phase A (5% HCO_2_H in H_2_O) and B (MeOH) used a gradient program at 4 mL/min, initially 95:5 (C:D), to 85:15 at 3 min, 75:25 at 13 min, 70:30 at 25 min, 45:55 at 35 min, 45:55 at 45 min, 5:95 at 46 min, 5:95 at 58 min, 95:5 at 60 min (Ferreres et al., 2003). Purified fractions were combined and freeze-dried before characterisation. Electrospray ionisation mass spectra (ESIMS) were recorded in positive ionisation mode on a VG Autospec spectrometer and in negative ionisation mode using the Micromass Quattro Ultima. NMR spectra were recorded using a Bruker Avance 500 MHz NMR spectrometer.

#### 4.7.1 6-*C*-β-Galactosyl-8-*C*-β-glucosylapigenin (**1**) (*D. incanum* root tissue)

HPLC RT = 14.30 min; UV (*λ*_max_ MeOH/H_2_O) 271, 336 nm. ^1^H NMR (500 MHz, d_4_-MeOH, 350 K) *δ* 8.02 (2H, *J *= 8.8, H2′, H6′), 6.96 (2H, d, *J *= 8.8, H3′, H5′), 6.66 (1H, s, H3). 6-*C*-β-Gal 4.99 (1H, d, *J *= 9.8 Hz, H1″), 4.05 (1H, t, *J *= 9.6, H2″), 4.03 (1H, m, H4″), 3.86–3.70 (2H, m, ^2^H6″), 3.72 (1H, m, H5″), 3.64 (1H, m, H3″). 8-*C*-β-Glu 5.05 (1H, d, *J *= 10.1, H1‴), 4.12 (1H, t, *J *= 9.5 Hz, H2‴), 3.97 (1H, dd, *J *= 1.9, 12.1 Hz, H6a″), 3.80 (1H, m, H6b″), 3.68 (1H, t, *J *= 9.2 Hz, H4‴), 3.56 (1H, t, *J *= 8.9, H3‴), 3.48 (1H, m, H5‴). ^13^C NMR (500 MHz, d_4_-MeOH, 350 K) *δ* 182.9 (C4), 165.3 (C2), 161.8 (C7), 161.4 (C4′), 159.0 (C5), 156.1 (C9), 128.6 (C2′, C6′), 122.1 (C1′), 115.6 (C3′, C5′), 107.6 (C6), 104.4 (C8), 104.1 (C10), 102.6 (C3). 6-C-β-Gal 74.5 (C1″), 70.5 (C2″), 74.4 (C3″), 69.2 (C4″), 79.6 (C5″), 61.6 (C6″). 8-C-β-Glu 74.0 (C1‴), 71.6 (C2‴), 78.9 (C3‴), 71.1 (C4‴), 81.4 (C5‴), 61.5 (C6‴). ESIMS *m/z* (CV = 160, rel. int.): 593 [M−H]^−^ (100), 503 [M−H−90]^−^ (9), 473 [M−H−120]^−^ (26), 383 [M−H−210]^−^ (33), 353 [M−H−240]^−^ (47).

#### 4.7.2 6,8-di-*C*-β-Glucosylapigenin (**2**) (vicenin-2) (*D. incanum* leaf tissue)

RT = 16.21 min; UV (*λ*_max_ MeOH/H_2_O) 272, 334 nm. ^1^H NMR (500 MHz, d_4_-MeOH, 350 K) *δ* 7.92 (2H, *J *= 8.4, H2′, H6′), 6.97 (2H, d, *J *= 8.4, H3′, H5′), 6.61 (1H, s, H3), 6-*C*-β-Glu 5.02 (1H, d, *J *= 9.9 Hz, H1″), 3.90 (1H, br, H2″), 3.89 (1H, dd, *J *= 2.6, 12.1 Hz, H6a″), 3.82 (1H, dd, *J *= 5.2, 12.1 Hz, H6b″), 3.57 (1H, t, *J *= 8.8, H4″), 3.56 (1H, m, H3″), 3.50 (1H, m, H5″). 8-*C*-β-Glu 5.10 (1H, d, *J *= 9.9, H1‴), 4.06 (1H, br s, H2‴), 3.94 (1H, dd, *J *= 2.5, 12.0 Hz, H6a″), 3.82 (1H, dd, *J *= 5.2, 12.1 Hz, H6b″), 3.65 (1H, t, *J *= 9.1 Hz, H4‴), 3.58 (1H, t, *J *= 8.8, H3‴), 3.52 (1H, m, H5‴). ^13^C NMR (500 MHz, d_4_-MeOH, 350 K) *δ* 182.8 (C4), 165.3 (C2), 161.6 (C7), 161.2 (C4′), 159.2 (C5), 155.9 (C9), 128.4 (C2′, C6′), 122.3 (C1′), 115.7 (C3′, C5′), 107.6 (C6), 103.7 (C8), 104.2 (C10), 102.8 (C3). 6-C-β-Glu 74.5 (C1″), 71.8 (C2″), 78.6 (C3″), 70.2 (C4″), 81.2 (C5″), 61.3 (C6″). 8-C-β-Glu 74.7 (C1‴), 71.9 (C2‴), 78.8 (C3‴), 70.7 (C4‴), 81.4 (C5‴), 61.5 (C6‴). ESIMS *m/z* (CV = 180, rel. int.): 593 [M−H]^−^ (100), 503 [M−H−90]^−^ (3), 473 [M−H−120]^−^ (7), 383 [M−H−210]^−^ (8), 353 [M−H−240]^−^ (10).

#### 4.7.3 6-*C*-β-Glucosyl-8-*C*-β-galactosylapigenin (**3**) (*D. incanum* root tissue)

RT = 16.72 min; UV (*λ*_max_ MeOH/H_2_O) 271, 335 nm. ^1^H NMR (500 MHz, d_4_-MeOH, 290 K) 8.27 (2H, d, *J *= 8.7 Hz, H2′, H6′), *δ* 7.92 (2H, *J *= 8.6, H2′, H6′), 6.95 (2H, d, *J *= 8.6, H3′, H5′), 6.92 (2H, d, *J *= 8.7, H3′, H5′), 6.74 (1H, s, H3), 6.71 (1H, s, H3). 8-*C*-β-Glu 5.05 (1H, d, *J *= 9.8 Hz, H1″), 4.93 (1H, d, *J *= 10.0 Hz, H1″), 4.36 (1H, br t, H2″), 3.66 (1H, br t, H2″), 3.91–3.71 (2 × 2H, m, H_2_6″), 3.59 (1H, m, H4″), 3.49 (1H, m, H4″), 3.56 (1H, m, H3″), 3.48 (1H, m, H3″), 3.51 (1H, m, H5″), 3.43 (1H, m, H5″). 8-*C*-β-Gal 5.12 (1H, d, *J *= 9.6, H1‴), 4.99 (1H, d, *J *= 10.0, H1‴), 4.44 (1H, t, *J *= 9.6 Hz, H2‴), 4.14 (1H, t, *J *= 9.2 Hz, H2‴), 4.09 (1H, d, *J *= 3.0 Hz, H4‴), 4.05 (1H, d, *J *= 2.4 Hz, H4‴), 3.91–3.71 (2 X 2H, H_2_6a″), 3.83 (1H, m, H5‴), 3.72 (1H, m, H5‴), 3.72 (1H, m, H3‴), 3.67 (1H, m, H3‴). ^13^C NMR (500 MHz, d_4_-MeOH, 290 K)⋅*δ* 182.9, 182.8 (C4), 165.6, 164.9 (C2), 162.0, 161.0 (C7), 161.3, 161.1 (C4′), 161.0, 159.6 (C5), 156.1, 154.6 (C9), 129.5, 128.4 (C2′, C6′), 122.8, 121.3 (C1′), 115.7, 115.5 (C3′, C5′), 108.2, 106.6 (C6), 106.5, 103.5 (C8), 104.2, 103.6 (C10), 102.4, 101.8 (C3). 6-C-β-Glu 74.7, 73.4 (C1″), 72.4, 70.6 (C2″), 78.8, 78.0 (C3″), 70.6, 69.6 (C4″), 81.6, 81.2 (C5″), 61.7–60.4 (C6″). 8-C-β-Gal 75.6, 74.0 (C1‴), 69.9, 69.1 (C2‴), 75.6, 74.7 (C3‴), 69.7, 69.0 (C4‴), 80.4, 79.7 (C5‴), 61.7–60.4 (C6‴). ESIMS *m*/*z* (CV = 150, rel. int.): 593 [M−H]^−^ (100), 503 [M−H−90]^−^ (7), 473 [M−H−120]^−^ (18), 383 [M−H−210]^−^ (16), 353 [M−H−240]^−^ (22).

#### 4.7.4 6-*C*-β-Galactosyl-8-*C*-β-arabinosylapigenin (**4**) (*D. incanum* root tissue)

RT = 18.44 min; UV (*λ*_max_ MeOH/H_2_O) 271, 336 nm. ^1^H NMR (500 MHz, d_4_-MeOH, 320 K) *δ* 8.09 (2H, br s, H2′, H6′), 6.96 (2H, d, *J *= 8.7 Hz, H3′, H5′), 6.67 (1H, s, H3), 6-*C*-β-Gal 4.98 (1H, d, *J *= 9.8 Hz, H1″), 4.21 (1H, br s, H2″), 4.03 (1H, d, *J *= 2.8 Hz, H4″), 3.84–3.75 (2H, m, H_2_6″), 3.72 (1H, m, H5″), 3.64 (1H, dd, *J *= 2.8, 9.4 Hz, H3″). 8-*C*-β-Ara 4.94 (1H, d, *J *= 9.8 Hz, H1‴), 4.44 (1H, t, *J *= 9.5 Hz, H2‴), 4.11 (1H, dd, *J *= 1.3, 12.6 Hz, H5a″), 4.04 (1H, br s, H4″), 3.80 (1H, br d, *J *= 13.8 Hz, H5b‴), 3.69 (1H, dd, *J *= 3.3, 9.2 Hz, H3‴). ^13^C NMR (500 MHz, d_4_-MeOH, 350 K) *δ* 182.5 (C4), 165.3 (C2), 161.6 (C7), 161.3 (C4′), 159.8 (C5), 155.6 (C9), 129.1 (C2′, C6′), 121.9 (C1′), 115.9 (C3′, C5′), 107.4 (C6), 104.0 (C8), 104.3 (C10), 102.6 (C3). 6-C-β-Glu 74.6 (C1″), 69.5 (C2″), 75.1 (C3″), 69.5 (C4″), 79.7 (C5″), 61.6 (C6″). 8-C-β-Ara 75.5 (C1‴), 69.3 (C2‴), 75.2 (C3‴), 69.5 (C4‴), 71.3 (C5‴). ESIMS *m*/*z* (CV = 180, rel. int.): 563 [M−H]^−^ (100), 473 [M−H−90]^−^ (10), 443 [M−H−120]^−^ (10), 383 [M−H−180]^−^ (14), 353 [M−H−210]^−^ (20).

#### 4.7.5 6-*C*-α-Arabinosyl-8-*C*-β-glucosylapigenin (**5**) (isoschaftoside) (*D. uncinatum*)

RT = 19.72 min; UV (*λ*_max_ MeOH/H_2_O) 271, 335 nm. ^1^H NMR (500 MHz, d_6_-DMSO, 300 K)⋅*δ* 7.95 (2H, *J *= 8.2 Hz, H2′, H6′), 6.91 (2H, d, *J *= 8.4 Hz, H3′, H5′), 6.64 (1H, s, H3), 6-*C*-β-Ara 4.65 (1H, d, *J *= 9.5 Hz, H1″), 4.03 (1H, br t, H2″), 3.82 (1H, d, *J *= 12.1 Hz, H5a″), 3.81 (1H, s, H4″), 3.59 (1H, d, *J *= 12.1 Hz, H5b″), 3.45 (1H, d, *J *= 8.9 Hz, H3″). 8-*C*-β-Glu 4.84 (1H, d, *J *= 9.7 Hz, H1‴), 3.92 (1H, t, *J *= 9.2 Hz, H2‴), 3.76 (1H, d, *J *= 11.5 Hz, H6a″), 3.55 (1H, dd, *J *= 5.6, 11.7 Hz, H6b″), 3.39 (1H, t, *J *= 8.9 Hz, H4‴), 3.33 (1H, t, *J *= 9.0, H3‴), 3.30 (1H, m, H5‴). ^13^C NMR (500 MHz, d_4_-MeOH, 350 K) *δ* 181.6 (C4), 163.5 (C2), 163.9 (C7), 161.1 (C4′), 158.8 (C5), 155.1 (C9), 126.8 (C2′, C6′), 121.9 (C1′), 114.0 (C3′, C5′), 108.5 (C6), 104.7 (C8), 102.3 (C10), 100.6 (C3). 6-C-β-Ara 72.8 (C1″), 67.5 (C2″), 72.6 (C3″), 67.1 (C4″), 68.4 (C5″). 8-C-β-Glu 72.2 (C1‴), 69.5 (C2‴), 77.4 (C3‴), 69.0 (C4‴), 80.1 (C5‴), 59.8 (C6‴). ESIMS *m*/*z* (CV = 150, rel. int.): 563 [M−H]^−^ (100), 503 [M−H−60]^−^ (1), 473 [M−H−90]^−^ (3), 443 [M−H−120]^−^ (3), 353 [M−H−210]^−^ (1).

#### 4.7.6 6-*C*-α-Arabinosyl-8-*C*-β-galactosylapigenin (**6**) (*D. incanum*)

RT = 21.10 min; UV (*λ*_max_ MeOH/H_2_O) 271, 337 nm. ^1^H NMR (500 MHz, d_4_-MeOH, 290 K) *δ* 8.27 (2H, *J *= 8.2 Hz, H2′, H6′), 6.92 (2H, d, *J *= 8.7 Hz, H3′, H5′), 6.70 (1H, s, H3), 6-*C*-β-Ara 4.90 (1H, d, *J *= 10.0 Hz, H1″), 4.10–4.05 (1H, m, H5a″), 4.01 (1H, t, 10.0 Hz, H2″), 4.01 (1H, br s, H4″), 3.80–3.71 (1H, m, H5b″), 3.67 (1H, dd, *J *= 3.1, 9.0 Hz, H4″). 8-*C*-β-Gal 5.00 (1H, d, *J *= 10.0 Hz, H1‴), 4.43 (1H, t, *J *= 9.6 Hz, H2‴), 4.10 (1H, br s, H4″), 3.98–3.80 (2H, m, ^2^H6″), 3.74 (1H, m, H5″), 3.68 (1H, dd, *J *= 3.7, 8.8 Hz, H3‴). ^13^C NMR (500 MHz, d_4_-MeOH, 350 K) 182.9 (C4), 165.3 (C2), 161.3 (C7), 161.3 (C4′), 159.5 (C5), 155.7 (C9), 129.3 (C2′, C6′), 121.6 (C1′), 115.5 (C3′, C5′), 106.8 (C6), 104.3 (C8), 104.2 (C10), 101.8 (C3). 6-C-β-Ara 75.0 (C1″), 69.4 (C2″), 73.8 (C3″), 69.4 (C4″), 70.5 (C5″). 8-C-β-Glu 74.1 (C1‴), 69.1 (C2‴), 75.5 (C3‴), 69.8 (C4‴), 80.4 (C5‴), 61.9 (C6‴). ESIMS *m*/*z* (CV = 150, rel. int.): 563 [M−H]^−^, 100), 473 [M−H−90]^−^ (15), 443 [M−H−120]^−^ (13), 383 [M−H−180]^−^ (39), 353 [M−H−210]^−^ (48).

#### 4.7.7 2″-*O*-Glucosyl-8-*C*-glucosylapigenin (**7**) (*D. uncinatum* root tissue)

RT = 20.15 min; UV (*λ*_max_ MeOH/H_2_O) 271, 334 nm. ^1^H NMR (500 MHz, d_6_-DMSO, 300 K) *δ* 8.20 (2H, *J *= 8.2 Hz, H2′, H6′), 6.95 (2H, d, *J *= 7.9 Hz, H3′, H5′), 6.73 (1H, s, H3), 6.28 (1H, s, H6), 6-*C*-β-Glu 4.86 (1H, d, *J *= 9.9 Hz, H1″), 4.13 (1H, t, *J *= 9.2 Hz, H2″), 3.75 (1H, d, *J *= 11.4, H6a″), 3.60 (1H, m, H6b″), 3.57 (1H, t, 9.5 Hz, H3″), 3.49 (1H, t, *J *= 9.1 Hz, H4″), 3.32 (1H, m, H5″). 2″-*O*-Glu 3.99 (1H, d, *J *= 7.8 Hz, H1‴), 3.22 (1H, d, *J *= 11.4 Hz, H6a‴), 3.12 (1H, d, *J *= 11.4 Hz, H6b‴), 2.98 (1H, m, H3‴), 2.98 (1H, m, H4‴), 2.83 (1H, t, *J *= 8.1 Hz, H2‴), 2.51 (1H, m, H5‴). ^13^C NMR (500 MHz, d_6_-DMSO, 300 K) 183.1 (C4), 165.0 (C2), 164.0 (C7), 162.4 (C4′), 162.0 (C5), 157.4 (C9), 129.6 (C2′, C6′), 122.8 (C1′), 116.7 (C3′, C5′), 105.0 (C8), 105.0 (C10), 103.3 (C3), 99.0 (C6). 6-C-β-Glu 72.3 (C1″), 81.7 (C2″), 79.2 (C3″), 71.0 (C4″), 82.5 (C5″), 61.3 (C6″). 2″-*O*-Glu 105.7 (C1‴), 75.2 (C2‴), 77.0 (C3‴), 70.3 (C4‴), 76.7 (C5‴), 61.9 (C6‴). ESIMS *m*/*z* (CV = 150, rel. int.): 593 [M−H]^−^ (100), 413 [M−H−180]^−^ (89), 293 [M−H−200]^−^ (24).

#### 4.7.8 8-*C*-Glucosylapigenin (**8**) (vitexin) (*D. incanum*)

RT = 26.43 min; UV (*λ*_max_ MeOH/H_2_O) 271, 349 nm. ^1^H NMR (500 MHz, d_6_-DMSO, 300 K)⋅*δ* 7.96 (2H, *J *= 8.4 Hz, H2′, H6′), 6.91 (2H, d, *J *= 8.5 Hz, H3′, H5′), 6.63 (1H, s, H3), 6.15 (1H, s, H6), 8-*C*-β-Glu 4.70 (1H, d, *J *= 9.8 Hz, H1″), 3.89 (1H, t, *J *= 9.0 Hz, H2″), 3.76 (1H, d, *J *= 11.4, H6a″), 3.55 (1H, dd, *J *= 5.7, 11.7 Hz, H6b″), 3.39 (1H, t, 9.3 Hz, H3″), 3.31 (1H, t, *J *= 8.5 Hz, H4″), 3.28 (1H, m, H5″). ^13^C NMR (500 MHz, d_4_-MeOH, 350 K) 181.6 (C4), 164.2 (C2), 164.0 (C7), 162.2 (C4′), 161.1(C5), 156.9 (C9), 129.3 (C2′, C6′), 122.5 (C1′), 116.7 (C3′, C5′), 105.7 (C8), 103.6 (C10), 103.0 (C3), 100.1 (C6). 6-C-β-Glu 74.8 (C1″), 72.2 (C2″), 79.8 (C3″), 71.6 (C4″), 82.6 (C5″), 62.4 (C6″). ESIMS *m*/*z* (CV = 150, rel. int.): 431 [M−H]^−^ (57), 341 [M−H−90]^−^ (11), 311 [M−H−120]^−^ (100).

## Figures and Tables

**Table 1:** Compounds characterised by isolation from tissues of *D. incanum* and *D. uncinatum* (denoted by A and B respectively).

**Figure:**
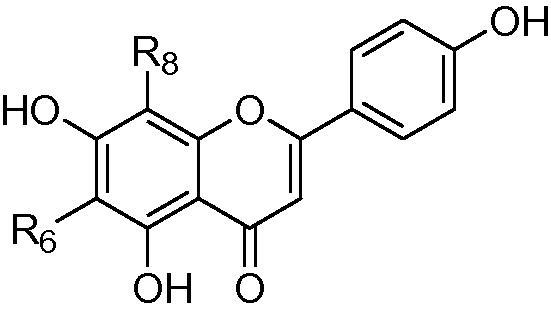

	R6	R8	LCMS retention time (min)
**1**^A^	*C*-β-d-galactose	*C*-β-d-glucose	13.73
**2**^A^	*C*-β-d-glucose	*C*-β-d-glucose	16.36
**3**^A^	*C*-β-d-glucose	*C*-β-d-galactose	16.66
**4**^A^	*C*-β-d-galactose	*C*-α-l-arabinose	18.10
**5**^B^	*C*-α-l-arabinose	*C*-β-d-glucose	20.15
**6**^A^	C-α-l-arabinose	*C*-β-d-galactose	21.46
**7**^B^	H	*C*-β-d-2″-*O*-glucosylglucose	25.03
**8**^B^	H	*C*-β-d-glucose	23.60
