# Supplementary material for: Isolation and identification of Desmodium root exudates from drought tolerant species used as intercrops against Striga hermonthica
Source: Phytochemistry. 2015 Sep;117:380–7. doi: 10.1016/j.phytochem.2015.06.026 (PMC4560159; doi:10.1016/j.phytochem.2015.06.026)
Supplement: Supplementary data [file mmc1.docx]

**4.6.1. 6-*C*-galactopyranosyl-8-*C*-glucopyranosylapigenin (1)**

Structure;

HPLC analysis;

14.30

LCMS analysis;

Low cone voltage MS;

High cone voltage MS;

^1^H NMR spectrum;





**4.6.2. 6-*C*-glucopyranosyl-8-*C*-glucopyranosylapigenin (2) Vicenin-2**

Structure;

HPLC analysis;

16.21

LCMS analysis;

Low cone voltage MS;

High cone voltage MS;

^1^H NMR spectrum;



**4.6.3. 6-*C*-glucopyranosyl-8-*C*-galactopyranosylapigenin (3)**

Structure;

HPLC analysis;

16.72

LCMS analysis;

Low cone voltage MS;

High cone voltage MS;

^1^H NMR spectrum;





**4.6.4. 6-*C*-galactopyranosyl-8-*C*-arabinopyranosylapigenin (4)**

Structure;

HPLC analysis;

18.436

LCMS analysis;

Low cone voltage MS;

High cone voltage MS;

^1^H NMR spectrum;





**4.6.5. 6-*C*-arabinopyranosyl-8-*C*-glucopyranosylapigenin (5) Isoschaftoside**

Structure;

HPLC analysis;

19.72

LCMS analysis;

Low cone voltage MS;

High cone voltage MS;

^1^H NMR spectrum;





**4.6.6. 6-*C*-arabinopyranosyl-8-*C*-galactopyranosylapigenin (6)**

Structure;

HPLC analysis;

21.10

LCMS analysis;

Low cone voltage MS;

High cone voltage MS;

^1^H NMR spectrum;

**

**

**4.6.7. 2”-*O*-glucosyl-8-*C*-glucosylapigenin (7)**

Structure;

HPLC analysis;

23.00

LCMS analysis;

Low cone voltage MS;

High cone voltage MS;

^1^H NMR spectrum;





**4.6.8. 8-*C*-glucopyranosylapigenin (8) Vitexin**

Structure;

HPLC analysis;

26.43

LCMS analysis;

Low cone voltage MS;

High cone voltage MS;

^1^H NMR spectrum;
